# Cancer Risks near Nuclear Facilities: The Importance of Research Design and Explicit Study Hypotheses

**DOI:** 10.1289/ehp.1002853

**Published:** 2010-12-10

**Authors:** Steve Wing, David B. Richardson, Wolfgang Hoffmann

**Affiliations:** 1 Department of Epidemiology, University of North Carolina–Chapel Hill, Chapel Hill, North Carolina, USA; 2 Institute for Community Medicine, Section Epidemiology of Health Care and Community Health, Ernst-Moritz-Arndt University of Greifswald, Greifswald, Germany

**Keywords:** childhood cancer, environmental epidemiology, ionizing radiation, methodology, nuclear power

## Abstract

**Background:**

In April 2010, the U.S. Nuclear Regulatory Commission asked the National Academy of Sciences to update a 1990 study of cancer risks near nuclear facilities. Prior research on this topic has suffered from problems in hypothesis formulation and research design.

**Objectives:**

We review epidemiologic principles used in studies of generic exposure–response associations and in studies of specific sources of exposure. We then describe logical problems with assumptions, formation of testable hypotheses, and interpretation of evidence in previous research on cancer risks near nuclear facilities.

**Discussion:**

Advancement of knowledge about cancer risks near nuclear facilities depends on testing specific hypotheses grounded in physical and biological mechanisms of exposure and susceptibility while considering sample size and ability to adequately quantify exposure, ascertain cancer cases, and evaluate plausible confounders.

**Conclusions:**

Next steps in advancing knowledge about cancer risks near nuclear facilities require studies of childhood cancer incidence, focus on *in utero* and early childhood exposures, use of specific geographic information, and consideration of pathways for transport and uptake of radionuclides. Studies of cancer mortality among adults, cancers with long latencies, large geographic zones, and populations that reside at large distances from nuclear facilities are better suited for public relations than for scientific purposes.

The possibility that radiation releases from nuclear facilities could cause cancer in surrounding populations has been of interest for more than two decades. Epidemiologic studies of spatial variation in cancer incidence or mortality have been conducted to investigate effects of unplanned releases as well as routine operations. For example, a case–control study of cancer among children < 5 years of age found that residence within 5 km of a nuclear facility was associated with a 61% [one-sided lower bound of the 95% confidence interval (CI), 26%] increased incidence of all cancer (Spix et al. 2008) and a 119% (lower bound of the 95% CI, 51%) excess risk of leukemia (Kaatsch et al. 2008a). A meta-analysis of geographic studies reported 23% (95% CI, 7–40%) higher incidence of leukemia among children 0–9 years of age living within 16 km of nuclear facilities (Baker and Hoel 2007). Other studies have compared risks among populations whose radiation doses have been estimated based on releases and transport of radiation or deposition of radionuclides. A study of thyroid disease among people who were exposed to radioactive iodine from the Hanford site in Washington State found that the risk of thyroid disease was similar regardless of the estimated doses from radioiodine (Davis et al. 2004), whereas a study of childhood leukemia after the Chernobyl accident, which classified radiation doses based on soil radioactivity and diet, reported an excess relative risk per gray of radiation of 32.4 (95% CI, 8.8–84.0) (Davis et al. 2006).

In April 2010 the U.S. Nuclear Regulatory Commission (NRC) asked the National Academy of Sciences (NAS) to analyze “radiogenic cancer mortality and total cancer mortality in populations living near past, present, and possible future commercial nuclear facilities for all age groups,” and to conduct the same analyses for cancer incidence (Sheron 2010). Nuclear power, weapons, and fuel-cycle plants are to be included. Before beginning the full study in late 2011, the NAS is to conduct a scoping study to determine availability of data, feasibility of considering geographic units smaller than counties, and the best study design for assessing risks. The NRC request underscores the need to evaluate logical problems with previous studies of cancer around nuclear facilities and to consider the appropriateness of specific hypotheses and design options. In the United States these issues are of interest, in part, because of continued nuclear weapons production and federal support for construction of new nuclear power plants.

Currently, the NRC relies on a 1990 report from the National Cancer Institute (NCI 1990) as its primary source for information about cancer risk from nuclear facilities (NRC 2010). That study compared cancer death rates in 107 counties that either contained, or neighbored a county that contained, a nuclear facility, with rates in 292 matched counties. For the period 1950–1984, investigators enumerated approximately 900,000 cancer deaths in nuclear facility counties and 1.8 million deaths in matched counties. A study of cancer incidence was restricted to Iowa and Connecticut, states that included four nuclear facilities. Jablon et al. (1991) summarized the findings from this study and concluded that “if nuclear facilities posed a risk to neighboring populations, that risk was too small to be detected by a survey such as this one.”

The NRC request for an “update” of the NCI study requires that NAS wrestle with several logical and methodological problems that have plagued the literature on cancer risks around nuclear facilities. Here we identify some key issues that must be addressed in order for the new study to advance science more than public relations.

## Hypothesis Formation and Research Design in Epidemiology

### General versus specific causation

Most epidemiologic studies investigate general exposure–response relationships; neither the source of exposure nor a particular population is of interest. A major consideration in such studies is that exposures and responses can be measured accurately. Populations that have been enumerated to evaluate the question of radiation and cancer include A-bomb survivors whose doses were estimated as a function of distance from hypocenter and shielding, patients exposed to medical or diagnostic radiation procedures recorded in clinical records, and workers whose occupational exposures have been monitored by individual dosimeters (National Research Council 2006). Results from general causation studies are often used to estimate risks in specific populations that have not, or cannot, be studied.

Other epidemiologic studies are designed to evaluate specific causation relevant to particular people, places, and times. Although hypotheses in these studies rely on knowledge of general causation, they aim to address the causes of disease in a particular population or similar populations. The question of cancer risks near nuclear facilities is specific because it concerns people who live near this category of facilities rather than the general exposure–response association for ionizing radiation and cancer. An even more specific question is about cancer risks near a particular nuclear facility (e.g., Hoffmann et al. 2007). The specificity of these questions necessitates focusing on one nuclear facility or groups of facilities even if quantifying exposures and responses in neighboring populations is difficult.

### Design of epidemiologic studies

Although usually nonexperimental, most epidemiologic studies are based on the model of an experiment in which subjects are randomized to be exposed or not, and all other conditions are kept identical in the two groups, including the assessment of responses. Although it is not necessary to know the mechanisms by which the exposure produces the response, knowledge about mechanisms is important for choosing factors to measure, measuring them correctly, and deciding the extent to which results support the hypothesis that the exposure causes the response. As in an experiment, sample size must be chosen so that the response occurs with sufficient frequency to permit comparison of the groups.

However, because exposures cannot be randomized in nonexperimental studies, large sample size does not provide confidence that other conditions that influence the response are similarly distributed in the exposed and unexposed groups, and these potential confounders must be considered in the data analysis and interpretation of results. Studies of cancer risks around nuclear facilities typically adjust for demographic factors that may differ between nearby populations and groups to which they are compared but do not collect information on other potential confounders.

### Descriptive versus analytic studies

Studies of disease trends and spatial patterns that do not focus on a specific etiologic agent are sometimes referred to as descriptive studies. Authors of some papers about cancer risks near nuclear facilities have labeled their studies descriptive, implying that they do not address a hypothesis (Laurier and Bard 1999; Laurier et al. 2008). However, studies of disease in populations surrounding a specific type of facility are of interest only if something released by that type of facility could cause the disease. Cancer risks near nuclear facilities are only of scientific interest because these facilities emit radiation and because ionizing radiation causes cancer. Calling a study descriptive does not remove the rationale for its conduct or reduce the importance of creating testable hypotheses about exposure and risk.

## Assumptions Required for Testable Hypotheses

An epidemiologic hypothesis might be that the response is higher in the exposed than the unexposed group. However, the scientific value of the hypothesis is not merely numerical; it depends on assumptions about the level of the exposure, the shape and magnitude of the exposure–response relationship, and the sample size, all of which affect the study power.

### Dose assumptions

A testable hypothesis requires a nontrivial difference in exposure between the groups being compared; the magnitude of difference that is nontrivial is a function of the dose response. Some studies of cancer around nuclear facilities have been conducted under the assumption that the exposure is too low to cause the response. For example, Jablon et al. (1991) quote U.K. researchers: “The increased occurrence of cancers in persons living near nuclear facilities could not have resulted from radioactive emissions from the facilities” because the doses were too low. Hatch et al. (1990) reported elevated cancer incidence in downwind areas after the 1979 radiation releases from the Three Mile Island unit 2 reactor but went on to study stress as an alternative explanation (Hatch et al. 1991) because radiation doses were “a fraction of the average U.S. exposure.” Kaatsch et al. (2008a), who reported elevated childhood cancer risk near German nuclear facilities, concluded, “The observed positive distance trend remains unexplained,” noting that “radiation exposure near German nuclear power plants is a factor of 1,000–100,000” below background. In a technical report they state that radiation must be excluded as a cause of the observed dose–response relationship on “fundamental grounds” (Kaatsch et al. 2008b).

All these authors assumed that radiation exposures were too small to cause a response. They did not expect to find positive relationships (Kaatsch et al. 1998). When they did, they could not conclude that the evidence supported rejection of the null hypothesis. British epidemiologist Geoffrey Rose described this situation in the Sellafield inquiry in the United Kingdom: “We were given information (which, it later transpired, was incorrect) of the total radioactive emissions from the plant, but the exposure levels of the children were a matter of speculation. The radiation experts on the committee calculated ‘best estimates’ and they concluded, on theoretical grounds, that these could not have caused any major excess risk: ‘It couldn’t have happened, so it didn’t happen’” (Rose 1991).

Assumptions about doses to populations near nuclear facilities are based on estimated releases, environmental dispersion, human uptake, and estimates of the relative biological effectiveness of different forms of radiation. Except in the case of short-term exposures during an accident, environmental assumptions involve average emission estimates, distances from facilities, and sometimes prevailing winds. Most epidemiologic studies of populations near nuclear facilities have not considered the spatial pattern of ingestion of radionuclides from food or water, nor have they measured radiation doses to individuals. All have been based on emission estimates that come from industries responsible for the releases and agencies responsible for regulating them.

### Dose–response assumptions

The consequence of assumptions about dose levels depends on another assumption, the dose response: the increase in cancer for each unit increase in radiation dose. When excess cancer near nuclear facilities cannot be interpreted as evidence of an effect of releases, it is because the expected response from the estimated dose is too small to detect. For example, authors of the Three Mile Island study cited an average whole-blood gamma dose in the range of 0.1–0.25 mSv in the 5-mile area around the plant (Hatch et al. 1990). The expected relative risk of cancer at this dose level, according to NAS BEIR V (Biological Effects of Ionizing Radiation, Health Effects of Exposure to Low-Level Ionizing Radiation) (National Research Council 1990) estimates available around the time of the study, which were primarily based on studies of acute penetrating radiation exposures of A-bomb survivors, would be less than 1.0005. Unless the dose estimates, the dose–response estimates, or both were considered to be questionable, and by a combined factor of orders of magnitude, no results from the study could have been interpreted as supporting the hypothesis that emissions caused cancer (Wing et al. 1997).

Past debate about obstetric X rays and childhood cancer illustrates the potential problem of overconfidence in the state of knowledge about a dose–response relationship. Although it is now widely assumed that the effect of fetal irradiation on childhood cancer risk is orders of magnitude higher (on a relative risk scale) than the effect of adult exposure (Wakeford 2008), early evidence that obstetric X rays cause childhood cancer (Stewart et al. 1956) was rejected, primarily based on studies of acute penetrating radiation exposures of A-bomb survivors. The Life Span Study of A-bomb survivors is important because of its large size and inclusion of females and males of all ages. However, the cohort was assembled 5 years after exposure, and cancer incidence data are not available until 12 years after exposure. There are no data for early childhood, the time period of most interest in studies of cancer risk near nuclear facilities. Difficulties of quantifying impacts of selective survival, dose misclassification, residual radiation, fallout, and other possible confounding factors on dose–response estimates suggest that caution should be used in extrapolating studies of acute radiation exposures in the Life Span Study to populations near nuclear facilities that may be chronically exposed to inhaled or ingested radionuclides.

### Study power: sample size and measurement of exposure and outcomes

The power or sensitivity of a study depends on the magnitude of the effect, the sample sizes in the exposure groups, and the ability to accurately measure exposures and outcomes. The weaker the relationship, the larger the sample size needed to detect it. If the effect of exposure is small, combining populations near multiple U.S. nuclear facilities is important for a study of cancer risks near nuclear facilities. However, if an exposure–response relationship does exist, it will be underestimated and may not be detected at all if people in the exposed and unexposed groups are mixed together. Large sample size is important, but when large sample size comes with poor exposure classification, the consequence is a statistically precise, biased estimate of effect.

Similarly, inability to track the response creates low study power. Assessment of cancer incidence (diagnosis) rather than death is important because many patients do not die of their cancers, and because the time between diagnosis and death increases the opportunity for people to move between communities with and without nuclear facilities (sometimes as a result of their diagnosis). However, the lack of cancer registries with catchment areas covering populations residing near most U.S. nuclear facilities during their entire operating history presents a serious barrier to studying risks for all facilities during their entire periods of operation.

## Next Steps in Research on Cancer Risks near Nuclear Facilities

Many studies of cancer near nuclear facilities have been conducted since the 1990 NCI study. An update of that study should build on what has been learned. Two recent childhood cancer studies have relatively large sample sizes: the meta-analysis of childhood leukemia in proximity to nuclear facilities conducted by Baker and Hoel (2007) and the Kinderkrebs in der Umgebung von Kernkraftwerken (KiKK) case–control study of childhood leukemia (Kaatsch et al. 2008a, 2008b) and childhood cancer (Spix et al. 2008) in the vicinity of German nuclear facilities. These studies are of particular interest because of the high radiosensitivity of the embryo, fetus, and infant, the use of incidence rather than mortality data, and the ability to discriminate populations in close proximity to nuclear reactors (Fairlie 2009a, 2009b, 2010; Nussbaum 2009). After intake, two radionuclides emitted by nuclear reactors, ^3^H (tritium in the form of heavy water) and ^14^C, are distributed throughout the body, and concentrations are 50–60% higher in fetal than in maternal tissues (Stather et al. 2002). Nuclear reactors routinely emit tritium and ^14^C, and spikes are observed during refueling (Fairlie 2010). From these observations, we suggest several key considerations for research on cancer risks near U.S. nuclear facilities.

### Exposure assessment

Studies of cancer risks around nuclear facilities under routine operations have focused on distance of residence from the facilities as the primary measure of exposure. Baker and Hoel (2007) focused on populations within 16 km (10 miles) of nuclear facilities. Studies based on large administrative districts, such as U.S. counties, including the 1990 NCI study (Jablon et al. 1991), do not have sufficient spatial specificity to produce meaningful findings.

The KiKK study compared the distance from the nearest nuclear facility of the residences of childhood cancer cases at the time of diagnosis and distances of residences of disease-free controls in high geographic resolution (100 m) (Kaatsch et al. 2008a; Spix et al. 2008). KiKK researchers analyzed risk as a continuous function with an *a priori* model of the reciprocal of distances ≥ 70 km, but the effects primarily reflect excesses in the vicinity of approximately 10 km of nuclear facilities. Several authors have emphasized the KiKK study’s precise distance measures as an advantage of the study (Fairlie 2010; Nussbaum 2009). Although such precision is desirable, the KiKK study did not analyze residence at birth or conception, which would be more relevant to fetal dose, nor did it evaluate residential history from conception to diagnosis, which would be relevant to exposure history. Other case–control studies should be designed to obtain such information.

However, residential distance is not a measure of dose, nor is it a good proxy unless all nuclear facilities have the same quantities and types of releases, pregnant mothers and children stay at home all the time, house construction and time outdoors do not affect exposure, and wind direction and diet are unimportant. These factors could be considered by conducting dose reconstructions based on environmental data for each facility and behavioral data from the populations being studied. This type of approach has been taken to a greater or lesser extent in some studies of single facilities (Davis et al. 2004, 2006; Hatch et al. 1990), but great effort and adequate data would be required to make such assessments for many facilities over long periods of time. An alternative strategy would be to classify exposure based on residential histories and to use mixed regression models to model the interfacility variability in distance–cancer relationships.

Measuring exposure during the correct time period is critical. Studies of young children have an advantage in this regard because the lag time between exposure and diagnosis of cancer is restricted compared with adults and there is less opportunity for children to change residences. Especially in studies of childhood cancer, the operations history of a facility must be considered. For example, a child diagnosed with cancer at 4 years of age who lived near a nuclear power plant that began operations 2 years earlier could not have experienced *in utero* exposure to emissions from that plant. Similarly, air emissions from an operating reactor could not affect a child diagnosed at 4 years of age if the plant ceased operation 5 years earlier, but drinking water contaminated by radionuclides with sufficient half-lives could be important from conception through the date of diagnosis. These scenarios underscore the need to consider time periods of operation, releases, environmental pathways, uptake, and internal doses, including the physical half-lives, environmental transformations, and biokinetics of radionuclides of interest. Such efforts have been made for studies of cancer risks near Chernobyl and Hanford (Davis et al. 2004, 2006), although not without problems (Hoffman et al. 2007).

### Outcome assessment

Studies of cancer risks near nuclear facilities should rely on incidence data; however, only mortality data are available nationally for the locations and time periods of operation of all nuclear facilities in the United States. Unlike some countries where this research question has been addressed, the United States lacks a medical insurance system that could be used to track cancer incidence nationally. States have instituted cancer registries at different times and with varying degrees of regional coverage and quality. A new study should be restricted to locations and time periods for which adequate cancer incidence data can be assembled. Additionally, because the ability to ascertain incident cancers among people who live near nuclear facilities declines with time and movement outside areas covered by state cancer registries, the short exposure lag for children improves the prospects for complete ascertainment of childhood cancers.

### Dose response

The inability of previous investigators to interpret positive findings as evidence in support of the hypothesis under investigation results, in part, from the belief that the dose response is too small to be detectable. One remedy for this problem is to select a sensitive subpopulation for investigation. In their meta-analysis, Baker and Hoel (2007) included only populations < 25 years of age, and they focused on children < 10 years of age. The KiKK study includes only children < 5 years of age. The focus on young ages is justified because of theory and evidence of greater risks from *in utero* and childhood than adult exposures, and because previous studies have found the strongest associations for children.

### Sample size

Childhood cancer occurs infrequently, so nuclear facilities with few children nearby cannot contribute many cases to an epidemiologic study. However, population size has little effect on the effort required to evaluate historical releases and environmental pathways. The most efficient expenditure of time and money would be to give priority to inclusion of facilities with larger nearby populations. Although population size is an important consideration, selection of facilities with larger nearby populations could be problematic if it led to systematic exclusion of facilities with larger estimated releases (Körblein and Hoffmann 1999).

### Potential confounders

Other causes of cancer could bias estimates of cancer risk from nuclear facilities if they are more or less common among populations around nuclear facilities than in comparison populations. One advantage of restricting a study to children is that they are less exposed to potentially confounding occupational and lifestyle carcinogens than are adults. Although the KiKK study did not achieve a high enough response rate among control children to use data on other cancer risk factors in primary analyses, ambient pesticide exposure, medical X rays (child and mother, diagnostic and therapeutic), fertility treatment, infections, medical drugs during pregnancy, and hair dye use were not associated with distance from nuclear power plants (Kaatsch et al. 2008b). Measurements of medical radiation, other sources of radiation, or other carcinogenic exposures, even if they are obtained from independent surveys, could be used to evaluate whether these factors are strongly enough correlated with nuclear facilities to result in an appreciable bias that could create or mask distance–cancer relationships observed in an epidemiologic study.

Although not yet identified, viruses may play a role in the development of childhood leukemia. Studies of time in day care during infancy, a measure of potential viral exposure, show protective effects for childhood leukemia (Petridou et al. 1993; Urayama et al. 2008), whereas studies of in-migration to rural areas, another possible source of viral exposure, suggest that population mixing increases risk (Kinlen et al. 1995; Wartenberg et al. 2004). A case–control study could obtain history of day-care exposures, and in-migration could be evaluated in either a case–control or area-based design.

Another method of evaluating confounding is to measure cancer incidence near nuclear facilities during the time period preceding startup. If one or more confounding factors, known or unknown, is associated with proximity, a relationship between proximity and cancer would be observed before startup. The prestartup dose–response estimate, which quantifies the degree of confounding under the assumption that the spatial distribution of the confounding factors is the same before and after startup, can then be subtracted from the poststartup dose response to control this source of bias (Hatch et al. 1990; Wing et al. 1997).

### A Bayesian perspective

One way to minimize problems of circular logic in the interpretation of epidemiological results (the null hypothesis cannot be rejected because we assume the exposure was too small to cause an effect), and to better inform power calculations for any future study, is to encourage investigators to explicitly state their prior beliefs. In a Bayesian framework, assumptions about dose and dose response are made explicit in prior distributions and then updated based on new evidence. If the investigators hold strong prior beliefs about the magnitudes of dose and the dose effects, then it may be helpful to recognize at the outset that a proposed study may have little ability to shift posterior estimates of effect. Then researchers could avoid conducting studies that have little ability to affect strong prior convictions about the association of interest.

## Conclusions

The NRC has asked the NAS to study mortality from all types of cancer, cancer at all ages, and cancer at sites where nuclear facilities might be licensed in the future. The considerations reviewed here suggest that such an approach could lead to an excessive number of comparisons. Effects in subgroups of interest could be discounted if considered in the context of a large number of extraneous comparisons. Fortunately, the NRC has also asked the NAS to evaluate radiation doses to off-site populations and to recommend the best epidemiologic study design.

The only scientific reason to conduct studies of cancer around nuclear facilities is to evaluate whether radiation doses to neighboring populations result in a detectable increase in cancer risk. It is not logical to test a hypothesis of elevated cancer near facilities if it is decided *a priori* that results cannot be interpreted as evidence in support of the hypothesis. Such an exercise would amount to a public relations effort masquerading as a scientific study. Authors of a study of doses from the 1979 radiation releases at Three Mile Island were explicit about the intent of their methodology, which they described as having been developed “for educational, public relations and defensive epidemiology purposes” (Gur et al. 1983). This is apparently the scenario that is envisioned by Ralph Andersen of the Nuclear Energy Institute in reference to the NRC’s request to the NAS: “These types of studies simply cannot even imply causality, and I would be disappointed if this study undertook to believe that it was a study of causality” [Andersen 2010; see Supplemental Material for audio recording of the 15th meeting of the Nuclear and Radiation Studies Board of the National Academies, Washington, DC, 26 April 2010 (doi:10.1289/ehp.1002853)].

On the contrary, we believe the only reason to conduct a study is to address causal hypotheses regarding cancer risks near nuclear facilities. To preserve the integrity of scientific research in this area, there must be careful engagement with issues of the physical and biological mechanisms of interest and selection of populations for study based on the ability to obtain adequate measurements and sample sizes.
